# Neutrophil to lymphocyte ratio might help prediction of acute myocardial infarction in patients with elevated serum creatinine

**DOI:** 10.12669/pjms.321.8712

**Published:** 2016

**Authors:** Ahmet Nalbant, Hakan Cinemre, Tezcan Kaya, Ceyhun Varim, Perihan Varim, Ali Tamer

**Affiliations:** 1 Dr. Ahmet Nalbant, Internal Medicine Consultant, Department of Internal Medicine, Sakarya University School of Medicine, Sakarya, Turkey; 2 Dr. Hakan Cinemre, Associate Professor, Department of Internal Medicine, Sakarya University School of Medicine, Sakarya, Turkey; 3 Dr. Tezcan Kaya, Assistant Professor, Department of Internal Medicine, Sakarya University School of Medicine, Sakarya, Turkey; 4 Dr. Ceyhun Varim, Assistant Professor, Department of Internal Medicine, Sakarya University School of Medicine, Sakarya, Turkey; 5 Dr. Perihan Varim, Cardiology Consultant, Dept. of Cardiology, Sakarya University School of Medicine, Sakarya, Turkey; 6 Prof. Dr. Ali Tamer, Department of Internal Medicine, Sakarya University School of Medicine, Sakarya, Turkey

**Keywords:** Acute myocardial infarction, Neutrophil to lymphocyte ratio, Renal insufficiency

## Abstract

**Background and Objective:**

Diagnostic performance of troponin assays is affected by renal insufficiency. Neutrophil to lymphocyte ratio(NLR) is an independent predictor of acute coronary syndrome. Our objective was to evaluate performance of NLR in diagnosing acute myocardial infarction (AMI) among patients with elevated serum creatinine.

**Methods:**

Patients with elevated creatinine levels evaluated for coronary artery disease were included (n=284). Patients were divided into two groups according to having AMI or non-specific chest pain. AMI diagnosis was made based on clinical and laboratory data, including serial EKG and cardiac enzymes, ECHO and coronary angiography.

**Results:**

Troponin, neutrophil, and NLR were found to be higher in patients with AMI, compared to patients without AMI (*P*= 0.001, *P*= 0.001 and *P*=0.028, respectively). ROC curve analysis for NLR in diagnosing AMI was significant (AUC: 0.607; *P*=0.003). Sensitivity, specificity, LR +, LR-, PPV and NPV for NLR>7.4 were found as 42.3%, 74.7%, 1.68%, 0.77%, 77% and 40%, respectively. Logistic regression analysis revealed that patients whose NLR>7.4 were 2.18 times as likely to have AMI.

**Conclusions:**

NLR can be used as an independent predictor of AMI in patients with renal insufficiency. This seems to get more important in the era of high sensitivity troponin assays. Our results might also help in early diagnosis of AMI in this high risk population while serial cardiac enzyme results are pending.

## INTRODUCTION

Cardiovascular events are the most prevalent reason of mortality in the patients having renal insufficiency.^1^ In patients with renal insufficiency, diagnosis of acute coronary syndrome through standard methods is not straightforward.^2^ Although elevated troponin-I(Tn-I) level is a significant diagnostic and prognostic indicator in predictingAMI^3^,^4^, its predictive performance decreases in case of elevated serum creatinine.

High Tn-I levels in patients with reduced renal function are likely to be multifactorial. Intact troponins are large molecules; therefore, the kidneys canprimarily be responsible for their clearance from the serum.^5^ Additionally, coronary ischemia is frequently present in patients with renal insufficiency and it is not very clear whether the Tn-I elevation is caused by ischemia or the chronic process of renal insufficiency. Although there is no specified method, increasing troponin levels during follow-up are interpreted in favor of acute coronary ischemia.^6^

Inflammation plays a significant role in myocardial ischemia. White blood cell count, neutrophil, lymphocyte count and NLR are among the indicators of systemic inflammation.^7^,^8^ They usually are accepted as significant indicators of inflammation in cardiovascular diseases as well.^9^ Neutrophils directly play role in the initiation of plaque rupture.^10^ In parallel, it is revealed that NLR is associated with left ventricular systolic function and acute coronary syndrome.^11^-^13^ It is reported that NLR is a predictor of the progress of coronary atherosclerosis in cases with coronary artery disease (CAD).^14^ It is also reported that NLR is a predictor of developing cardiac heart failure after myocardial infarction and of mortality in ST-segment elevated myocardial infarction.^15^

In accordance with these data, we designed this study to investigate whether NLR has a role in making the diagnosis of AMI in the patients with high creatinine levels.

## METHODS

### Patients

There were 25,492 patients older than 18 years presented/admitted with chest pain to the emergency department, internal medicine, nephrology, cardiology clinics or coronary intensive care unit of our university hospital from January 2013 to October 2014. For the initial case selection purposes among these patients for impaired renal function, serum ceratinin >1.2 was used as the cut-off. After initial selection, 1,482 patients with serum creatinine > 1.2mg/dL were left. Exclusion criteria were chest pain due to etiologies other than AMI, active infection, autoimmune diseases, malignancies, liver disease such as hepatitis and cirrhosis, pregnancy, hematological disease, cerebrovascular disease and trauma. After the exclusion process 284 patients with typical chest pain and Tn-I was ≥ 0.1 ng/ml were left. Their mean serum creatinin was 1.7 mg/dL. These 284 patients who were further investigated for AMI were included in the study. This study was approved by Sakarya University School of Medicine Ethical Committee for Clinical Research(No. 71522473/050.01.04/11; Jan 21, 2015).

### Evaluation

Cardiology department determined whether a patient had AMI or not according to the criteria recommended by American College of Cardiology.^16^ Serum neutrophil, lymphocyte, platelet, and mean platelet volume (MPV) data of all patients were recorded and NLR and platelet/lymphocyte ratio (PLR) values were calculated. Patients were categorized into two groups as AMI and non AMI. Serum creatinine was measured with the architect C 16000 (Abbott) device by using the kinetic alkaline picrate method at the biochemistry laboratory of the hospital. On the other hand, Tn-I was examined with the Architect I 2000SR device by using chemiluminescence (ECLIA) method. Reference range of creatinine was 0.72- 1.19 mg/dl and Tn-I was 0.00 - 0.033 ng/ml. Hemogram parameters were measured by using Cell-dyn 3700.

### Statistical Analysis

Data analysis was performed by using statistical software (SPSS, version 10.0 [SPSS Inc, Chicago, IL]. Normally distributed data were compared via one-way analysis of variance, and non–normally distributed data were compared via Mann- Whitney U test. Categorical associations were evaluated by using χ^2^ test and multiple logistic regression. Goodness of fit was determined by using Nagelkerke R^2^ and Hosmer-Lemeshow goodness-of-fit test. Performance of the diagnostic test was assessed using receiver operating characteristic (ROC) curve analysis and with calculation of area under the curve (AUC) of the ROC curves. Statistical significance was defined by *P*≤ .05.

## RESULTS

**Fig.1:**
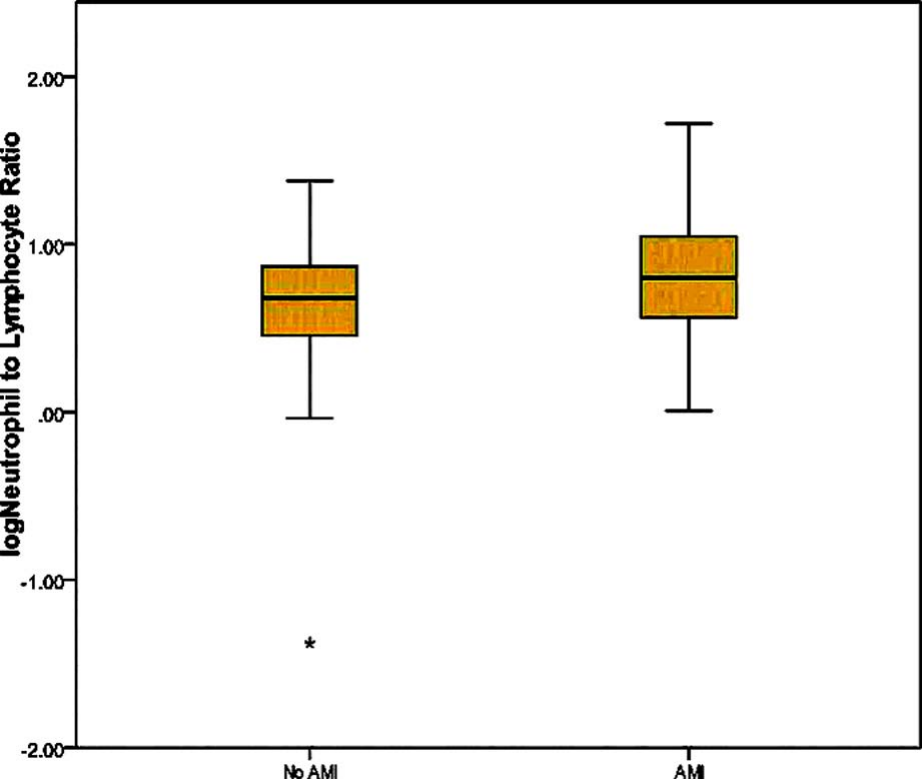
Mean neutrophil to lymphocyte ratio in patients with and without acute myocardial infarction.

There were 181 male and 103(36%) female patients. Table-I illustrates demographic and laboratory characteristics of patients. AMI was diagnosed in 189(66.5%) of 284 patients. Of these,71 patients had ST elevated and 118 patients had non-ST elevated AMI. The distribution of ST elevated AMI was as follows: Inferior wall 32, anteroseptal 30, high lateral 4, anteroseptal+ inferior wall 1. There were 8 patients on chronic hemodialysis. Mean(SD) age was 70.2(11.3) in patients with AMI and was 70.5(13.5) in those without AMI which did not differ significantly (*P*=0.872). There was also no difference between two groups in terms of gender (χ^2^ = 0.106, *P*=0.745). Tn-I levels were significantly higher in patients with AMI compared to patients without AMI (*P*<0.001). Creatinine was significantly higher in patients without AMI compared to patients with AMI (*P*<0.001). Neutrophil count and NLR were significantly higher in patients with AMI compared to patients without AMI (*P*<0.001 and *P*=0.003, respectively). There was no difference between the cases with and without AMI in terms of platelet, lymphocytes, MPV and PLR (Table-II). Mean NLR in patients with and without AMI is shown in Fig.1.

**Table-I:** Demographic and laboratory characteristics of patients with elevated troponin and creatinine (n =284).

	Female (n =103)	Male (n =181)	P
Age, years*	73.7(11.3)	68.5(12.1)	<0.001
Platelet count, ×1000/µL**	223 (86)	220 (91)	0.929
Mean platelet volume, fL**	8.5(1.4)	8.1(1.6)	0.047
Troponin I, ng/ml**	3.2(7)	3.5 (18)	0.199
Creatinine, mg/dl**	1.8 (2.2)	1.7 (1.2)	0.193
Neutrophil to lymphocyte ratio**	5.3 (5.1)	6 (7.8)	0.414
Neutrophil count, ×109/L**	7.3 (4.6)	8.1 (5.9)	0.030
Lymphocyte count, ×109/L**	1.4 (1.1)	1.5 (1.2)	0.444
Platelet to lymphocyte ratio**	165 (135)	147 (130)	0.442

* Mean (Standard Deviation)

** Median (Interquartile Range)

**Table-II:** Demographic and laboratory characteristics of patients with and without acute myocardial infarction (AMI).

	With AMI (n = 189)	Without AMI (n = 95)	P
Gender, male/female	119/70	62/33	0.704
Age, years*	70.2(11.3)	70.5(13.5)	0.872
Neutrophil to lymphocyte ratio**	5.58(6.6)	5.1(7.6)	0.003
Neutrophil count, ×109/L**	8.0(5.5)	6.3(4.3)	<0.001
Lymphocyte count, ×109/L	1.5(1)	1.3(0.9)	0.178
Platelet count, ×1000/µL**	221(100)	215(122)	0.168
Mean platelet volume, fL**	7.9(1.9)	7.8(2.1)	0.138
Platelet to lymphocyte ratio**	151.4(133.4)	153(137.6)	0.998
Troponin-I, ng/ml**	3.2(13)	0,42(0.9)	<0.001
Creatinine, mg/dl**	1.7(2)	2.8(2.7)	<0.001

* Mean (Standard Deviation)

** Median (Interquartile Range)

**Fig.2:**
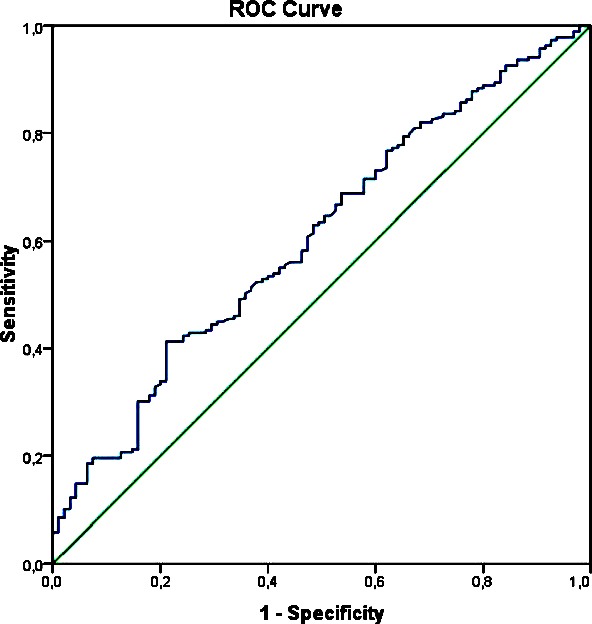
Receiver operating characteristic curve for neutrophil to lymphocyte ratio in patients with or without acute AMI.

The effect of NLR on making the diagnosis of AMI was analyzed by ROC curve and AUC was significant ((AUC:0.607; *P*=0.00, 95% CI 0.538to 0.675). (Fig.2). Sensitivity, specificity, positive predictive value, negative NPV, LR+, LR- values and the disease prevalence for NLR> 7.4 were 42.3%, 74.7%, 77%, 40%, 1.7, 0.8 and 66.5%, respectively. Logistic regression analysis using NLR >7.4 as categorical and serum creatinine as continuous variable revealed that patients with NLR> 7.4 were 2.184 times as likely to have AMI(B= 0.781, Standart Error= 0.290, Wald= 7.270, Odds Ratio= 2.184, P= 0.007 for NLR and B= -0.282, Standart Error= 0.068, Wald=17.474, Odds Ratio=0.754, P=0.001 for serum creatinine). Nagelkerke R^2^ was 13.8%.

## DISCUSSION

In this study, NLR and neutrophil count were found to be significantly higher in the patients with AMI and renal insufficiency compared to the patients without AMI. On the other hand, serum creatinine was significantly higher in the non-MI group. In terms of gender, age, platelet, lymphocyte, MPV and PLR there was no significant difference between patients with and without AMI. We found that patients with NLR > 7.4 were 2.18 times as likely to have AMI.

It is known that inflammation has significant role in all stages of atherosclerosis.^17^ It is also reported that the increase in neutrophil count in patients with ST elevated myocardial infarction is associated with short-term prognosis and infarct size and neutrophil mediate the inflammatory response resulting from acute myocardial damage.^18^ Our finding of elevated neutrophil counts in patients with AMI in this study agreed with these reports.

Various studies have shown that there is an association between CAD and NLR. NLR is accepted as a parameter showing lymphocyte reduction and physiological stress together and reflecting acute inflammation.^19^ Kalay et al. showed that high NLR in patients with acute coronary syndrome is an independent predictor for development of coronaryatherosclerosis.^14^ In our study we found significantly increased NLR in patients with AMI and this agreed with these reports. However, there was no significant difference in lymphocyte counts in our study. Thus, NLR elevation was likely to be associated with increase in the neutrophil count rather than a decrease in lymphocytes.

It is known that platelets, MPV, and PLR change significantly in acute coronary events.^20^ MPV is a significant indicator of platelet activation and function.^21^ However in our study, we could not find an association of platelet count, MPV, and PLR with AMI. This might be due to studying among patients with renal insufficiency.

Predictive role of Tn-I decreases in the presence of impaired renal function. Previous studies have shown that Tn-I can be falsely elevated in the patients with renal insufficiency or in hemodialysis patients.^22^,^23^ Khan et al., conducted a study on 102 patients to determine the diagnostic significance of Tn-I as a cardiac marker in the presence of a broad spectrum of diseases and found nonspecific elevation of Tn-I in patients with a variety of disorders including renal insufficiency.^24^ Newly introduced high sensitive Tn-I assays is likely to increase this problem.^25^ Tn-I was used in diagnosing of AMI also in our study together with other tests like coronary angiography and hence we think that our finding of lower serum creatinine in AMI patients was due to increased specificity of the test with lower creatinine values. This was also revealed by a significant effect and a negative coefficient for creatinine in the logistic regression analysis.

### Limitations of the study

Because of the methodology, our study has been a subgroup analysis. We couldn’t evaluate all of the cratinine elevated 1,482 patients for AMI in detail and selected 284 patients with typical chest pain and Tn-I >0.1 among them. This troponin cut-off has already been used by our cardiology department in patients with impaired renal function and there had negligible number of patients ruled in for AMI later on in their clinical practice for the last 10 years. Finally, we appreciate that Tn-I is a cardiac specific test and our intent was not to introduce an alternative to Tn-I. Our aim was to study NLR in patients with impaired renal function and to show whether it might provide help in the cases where the diagnosis is not straight forward.

## CONCLUSION

To the best of our knowledge, this is the first study showing the predictive value of NLR in AMI among patients with renal insufficiency. We found that NLR was significantly elevated in AMI among this patient population. We also provided a cut-off value for this readily available test in this patient group and showed that patients with NLR>7.4 were 2.18 times as more likely to have AMI compared to patients whose NLR was ≤7.4. Our results might also help in early diagnosis of AMI in these high risk patients while serial cardiac enzyme measurements are pending. With the introduction of high sensitivity troponin with decreasing positive predictivity for the sake of increased sensitivity, such a help might become more important in the near future.

## References

[1] Foley RN, Parfrey PS, Harnett JD, Kent GM, Martin CJ, Murray DC (1995). Clinical and echocardiographic disease inpatients starting end-stage renal therapy. Kidney Int.

[2] Roppolo LP, Fitzgerald R, Dillow J, Ziegler T, Rice M, Maisel A (1999). A comparison of troponin T and troponin I as predictors of cardiac events in patients undergoing chronic dialysis at a Veteran's Hospital: a pilot study. J Am Coll Cardiol.

[3] Antman EM, Tanasijevic MJ, Thompson B, Schactman M, McCabe CH, Cannon CP (1996). Cardiac-specific troponin I levels to predict the risk of mortality in patients with acute coronary syndromes. N Engl J Med.

[4] Braunwald E, Antman EM, Beasley JW (2000). ACC/AHA guidelines for the management of patients with unstable angina and non-ST segment elevation myocardial infarction. A report of the American College of Cardiology/American Heart Association Task Force on Practice Guidelines. J Am Coll Cardiol.

[5] Newby LK, Jesse RL, Babb JD, Christenson RH, De Fer TM, Diamond GA, Fer TM (2012). ACCF 2012 Expert Consensus Document on Practical Clinical Considerations in the interpretation of Troponin Elevations. J Am Coll Cardiol.

[6] Van Lente F, McErlean ES, DeLuca SA, Peacock WF, Rao JS, Nissen SEK (1999). Ability of troponins to predict adverse outcomes in patients with renal insufficiency and suspected acute coronary syndromes: a case matched study. J Am CollCardiol.

[7] Zahorec R (2001). Ratio of neutrophil to lymphocyte counts rapid and simple parameter of systemic inflammation and stress in critically ill. Bratisl Lek Listy.

[8] Gillum RF, Mussolino ME, Madans JH (2005). Counts of neutrophils, lymphocytes, and monocytes, cause-specific mortality and coronary heart disease: the NHANES-I epidemiologic follow up study. Ann Epidemiol.

[9] Horne BD, Anderson JL, John JM, Weaver A, Bair TL, Jensen KR (2005). Intermountain Heart Collaborative Study Group. Which white blood cell subtypes predict increased cardiovascular risk?. J Am Coll Cardiol.

[10] Naruko BT, Ueda M, Haze K, van der Wal AC, van der Loos CM, Itoh A (2002). Neutrophil infiltration of culprit lesions in acute coronary syndromes. Circulation.

[11] Bekler A, Erbag G, Sen H, Gazi E, Ozcan S (2015). Predictive value of elevated neutrophil-lymphocyte ratio for left ventricular systolic dysfunction in patients with non ST-elevated acute coronary syndrome. Pak J Med Sci.

[12] Tamhane UU, Aneja S, Montgomery D, Rogers EK, Eagle KA, Gurm HS (2008). Association between admission neutrophil to lymphocyte ratio and outcomes in patients with acute coronary syndrome. Am J Cardiol.

[13] Uthamalingam S, Patvardhan EA, Subramanian S, Ahmed W, Martin W, Delay M (2011). Utility of the Neutrophil to Lymphocyte Ratio in Predicting Long-Term Outcomes in Acute Decompensated Heart Failure. Am J Cardiol.

[14] Kalay N, Dogdu O, Koc F, Yarlioglues M, Ardic I, Akpek M (2012). Hematologic para- meters and angiographic progression of coronary atherosclerosis. Angiology.

[15] Ghaffari S, Nadiri M, Pourafkari L, Sepehrvand N, Movasagpoor Rahmanyand N (2014). The predictive Value of Total Neutrophil Count and Neutrophil/Lymphocyte Ratio in Predicting In-hospital Mortality and Complications after STEMI. J Cardiovasc Thorac Res.

[16] Thygesen K, Alpert JS, Jaffe AS (2012). Third universal definition of myocardial infarction. J Am Coll Cardiol.

[17] Libby P, Ridker PM, Maseri A (2002). Inflammation and atherosclerosis. Circulation.

[18] Kirtane AJ, Bui A, Murphy SA, Barron HV, Gibson CM (2004). Association of peripheral neutrophilia with adverse angiographic outcomes in ST-elevation myocardial infarction. Am J Cardiol.

[19] Gibson PH, Cuthbertson BH, Croal BL, Rae D, El-Shafei H, Gibson G (2010). Usefulness of neutrophil/lymphocyte ratio as predictor of new onset atrial fibrillation after coronary artery bypassgrafting. Am J Cardiol.

[20] Ergelen M, Uyarel H, Altay S (2014). Predictive Value of Elevated neutrophil to lymphocyte ratio in patients undergoing primary angioplasty for ST-segment elevation myocardial infarction. Clin Appl Thromb Hemost.

[21] Thompson CB, Jakubowski JA (1988). The pathophysiology and clinical relevance of platelet heterogeneity. Blood.

[22] Freda BJ, Tang WH, Van Lente F, Peacock WF, Francis GS (2002). Cardiac troponins in renal insufficiency: Review and Clinical implications. J Am Coll Cardiol.

[23] Musso P, Cox I, Vidano E, Zambon D, Panteghini (1999). Cardiac troponin elevations in chronic renal failure: prevalence and clinical significance. ClinBiochem.

[24] Khan IA, Tun A, Wattanasauwan N, Win MT, Hla TA, Hussain A (1999). Elevation of serum cardiac Troponin I in noncardiac and cardiac diseases other than acute coronary syndromes. Am J Emerg Med.

[25] Pfortmueller CA, Funk GC, Marti G, Leichtle AB, Fiedler GM, Schwarz C (2013). Diagnostic performance of high-sensitive troponin T in patients with renal insufficiency. Am J Cardiol.

